# GPX4 is a key ferroptosis biomarker and correlated with immune cell populations and immune checkpoints in childhood sepsis

**DOI:** 10.1038/s41598-023-32992-9

**Published:** 2023-07-13

**Authors:** Guoxin Qu, Hui Liu, Jin Li, Siyuan Huang, Nannan Zhao, Ling Zeng, Jin Deng

**Affiliations:** 1 The First Affiliated Hospital of Hainan Medical University, Hainan Medical University, Haikou, 570100 People’s Republic of China; 2 The Affiliated Hospital of Guizhou Medical University, Guizhou Medical University, Guiyang, 550001 People’s Republic of China; 3 State Key Laboratory of Trauma, Burns and Combined Injury, Research Institute of Surgery, Daping Hospital, Army Medical University, Chongqing, 400042 People’s Republic of China

**Keywords:** Cell biology, Computational biology and bioinformatics, Immunology, Molecular biology, Biomarkers, Diseases

## Abstract

Sepsis is the uncontrolled reaction of the body to infection-induced inflammation, which results in life-threatening multiple-organ dysfunction (MODS). Although the research on sepsis has advanced significantly in recent years, its pathophysiology remains entirely unknown. Ferroptosis is a new-fashioned type of programmed cell death that may have an impact on sepsis development. However, the precise mechanism still needs to be explored. In this paper, Four pediatric sepsis datasets [training datasets (GSE26378 and GSE26440) and validation datasets (GSE11755 and GSE11281)] were chosen through the GEO (Gene Expression Omnibus) database, and 63 differentially expressions of ferroptosis-relation-genes (DE-FRGs) were eventually discovered using bioinformatics investigation. Functional annotation was performed using GO and KEGG pathway enrichment analysis. Then, four Core-FRGs (FTH1, GPX4, ACSL1, and ACSL6) were extracted after the construction of the protein–protein interaction (PPI) network and the research of the MCODE module. Consequently, Hub-FRG (GPX4) was found using the validation datasets, and correlation exploration of immunity populations (neutrophils, r =  − 0.52; CD8 T-cells, r = 0.43) and immunity checkpoints (CD274, r =  − 0.42) was implemented. The usefulness of GPX4 as a marker in sepsis was assessed in a mouse model of sepsis. The findings demonstrate that GPX4 is a crucial biomarker and a new latent immunotherapy target for the prediction and therapy of pediatric sepsis.

## Introduction

Sepsis is defined as the body's uncontrolled inflammatory response to infection, which causes various organ failures and endangers life^1^. Sepsis has a high incidence rate and fatality rate^2^. Every day, almost 14,000 individuals worldwide die from sepsis. More importantly, the incidence of sepsis in children is the highest, 40% of occurrences involved kids under the age of five, in comparison to other populations. The majority of children who died of sepsis lived in impoverished communities^3,4^. Furthermore, treating sepsis requires a large number of medical resources, constitutes a significant threat to human health, and cause a detrimental influence on life satisfaction^4^. Sepsis pathogenesis is exceedingly complicated. Previous studies focused on the associated inflammatory immune response and made some headway, but clinical trial research has not given a sufficient breakthrough. As a result, the research on sepsis remains challenging and critical.

**Figure 1:**
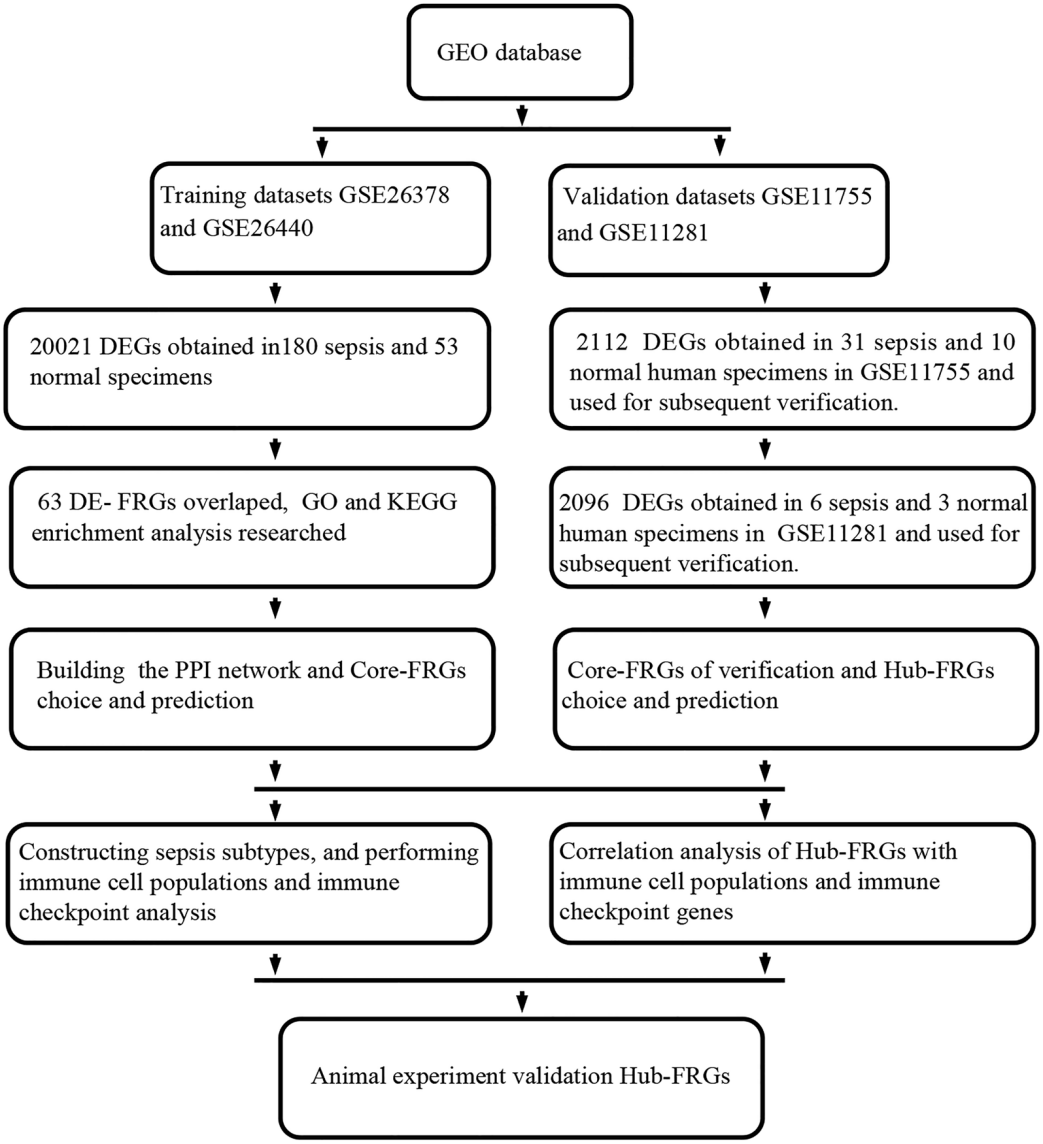
This study's flow diagram. GEO, Gene Expression Omnibus database; DEGs, differentially expressed genes; DE-FRGs, differentially expressed ferroptosis-related genes; GO, Gene Ontology; KEGG, Kyoto Encyclopedia of Genes and Genomes; PPI, protein–protein interaction.

Ferroptosis is a novel concept in biology that has recently become a focus of the scientific investigation. It is a distinct sort of iron dependent cell death programmed from apoptosis, necrosis, and autophagy^5^. Ferroptosis is primarily caused by the inactivation or reduction of glutathione peroxidase 4 (GPX4) production in cells, which results in a rise of lipid reactive oxygen species (ROS) and, ultimately, cell death^6–8^. In terms of cell shape, ferroptosis results in smaller mitochondria, increased membrane density and rupture, and decreased crista^9^. Recent research has discovered that a severe stress reaction in the septic patient can result in abnormal metabolism of ions, fats, and energy in the body^10^, and an imbalance in iron metabolism is directly associated with the pathophysiology of sepsis^11^. Hepcidin elevation was associated with a lower risk of death from sepsis^12,13^. Prauchner CA found that the accumulation of ROS was critical in the beginning and progression of various organ function impairments in sepsis^14^. Previously, it was commonly observed in the research related to tumor and ferroptosis, whereas ferroptosis and childhood sepsis research was seldom reported. As a result, more study into the molecular regulation and mechanism of ferroptosis in the progression of childhood sepsis is urgently needed. This study takes the connection between ferroptosis and childhood sepsis as a breakthrough point, obtains the data sets from the GEO, conducts bioinformatics analysis, screens out Hub-FRGs, and carries out the correlation analysis of their immune cell populations and immune checkpoints and biological verification, in order to provide a new biomarker of prediction value, potential immunotherapeutic target, and theoretical basis for the research of childhood sepsis (Fig. 1).

## Materials and methods

### Acquisition of the Pediatric Sepsis Dataset and the Ferroptosis-Related Genes (FRGs)

The GEO databank (http://www.ncbi.nlm.nih.gov/geo/), where the serial codes for the dataset for sepsis patients are GSE26378, GSE26440, GSE11755, and GSE11281, was utilized to obtain the data. As bioinformatics training cohorts, GSE26378 (n = 82 sepsis; n = 21 controls) and GSE26440 (n = 98 sepsis; n = 32 controls) comprised whole blood gene expression data from 180 pediatric sepsis patients and 53 normal kid samples. The validation cohorts were GSE11755 (n = 31 pediatric sepsis and n = 10 controls) and GSE11281 (n = 6 pediatric sepsis and n = 3 controls). The MolecularSignatures Databank (MSigDB) (https://www.gsea-msigdb.org/gsea/msigdb/)^15^ was used to obtain a ferroptosis gene dataset. After deleting duplicates, 65 experimentally verified ferroptosis-related genes (FRGs) were found for further investigation.

### Identification of DE-FRGs

The annotate package was used to annotate the probe ID with the gene name. Use the ComBat strategy (R-package: sva; ComBat) to eliminate the potential batch influence in GSE26378 and GSE26440. For background correction, the normal exponential convolution model was employed with the neqc function (R package: lima (version 3.40.6); Function: neqc) to normalize and convert each sample's strength^16^. To merge the data sets, use the R tool inSilicoMerging^17^ and compare the UMAP and boxplot of data sets prior to and following normalizing and de batch correction. The "annotation probe" software was employed to re-annotate the sequence matrix. The microarray-data were calibrated using the R software's "limma" tool, and DEGs were detected between septic patients and healthy controls, log2FC ≥ 1, adjusted *P* value < 0.05. The "ggplot2" software (V. 3.3.4) was utilized to display the DEGs in the volcano plot. Then, the differentially expressed FRGs (DE-FRGs) were generated by crossing DEGs and FRGs and displayed in the Venn-plot created with the 'Venn diagram' tool. The expression of the DE-FRGs was shown using the "ggplot2" software in heatmaps and multi-set boxplots.

### DE-FRGs functional annotation and pathway enrichment

The c5.go.mf.v7.gmt subset was derived in the MSigDb (https://doi.org/10.1093/bioinformatics/btr260, http://www.gsea-msigdb.org/gsea/downloads.jsp). To learn more about the probable roles of DE-FRGs, we used the ClusterProfiler (V. 3.14.1) software to analyze the Gene Ontology (GO)^18^ and Kyoto Encyclopedia of Genes and Genomes (KEGG)^19^ pathway enrichment. GO is a computer-based bioinformatics software that is mostly used to annotate genes and research their biological processes. KEGG is a computer statistical resource database that evaluates high-standard biological processes and function systems from a wide range of molecular datasets and discovers pathways in which DEGs may play a major role. The bubble diagram depicts the results of the enrichment. When the minimal gene array was 5, and the maximal gene array was 5000, FDR < 0.1 and Adjusted *P* < 0.05 were accepted as significant statistically.

### Construction of a protein–protein interaction network (PPI) for DE-FRGs

To examine the interaction between DE-FRGs, utilize the STRING database (Search Tool for the Retrieval of Interacting Genes, http://string-db.org/)^20^, and then use Cytoscape software 3.9.1 (http://cytoscape.org/)^21^ to construct and display PPI networks. Use the MCODE plug-in to explore the modules that are closely associated with genes for further investigation.

### Acquisition of core-FRGs

1136 mitochondrial-function-relation-genes (MFRGs) were obtained from the MitoCarta 3.0 online database (https://www.broadinstitute.org/mitocarta)^22^. The overlapping genes of DEGs and MFRGs were defined as the differentially expressed MFRGs (DE-MFRGs). The overlap genes of DE-MFRGs and MCODE-FRGs were named as "core differentially expressed FRGs" (Core-FRGs), to be verified later.

### Expression of core-FRG in the validation datasets

The Core-FRGs were validated using two sepsis datasets (GSE11755: n = 31 meningococcal sepsis, n = 10 Control; GSE11281: n = 6 Staphylococcus sepsis, n = 3 Control). The expression of the Core-FRGs was depicted in the volcano plot by the "ggplot2" software.

### Diagnostic value analysis of hub-FRG in the sepsis

The receiver operating characteristic curve (ROC) technique in the Python package was executed to analyze hub-FRG diagnostic effectiveness according to the training set and validation set.

### Consensus clustering analysis

Cluster analysis was done using ConsensusClusterPlus^23^ to generate clustering based on the "pam" approach in order to further understand the biologic characteristics modulated by FRGs in the sufferer's peripheral blood. The samples were repeated ten times, with an 80% resampling rate. The empirical cumulative distribution function plot was adopted to find the ideal clustering results. Then, the UMAP package (version 0.2.7.0) was employed for obtaining the reduced dimension matrix^24^ and evaluating DE-FRGs expression patterns in distinct subpopulations of peripheral blood.

### Gene set enrichment analysis (GSEA)

GSEA (v. 4.0) tool was acquired from GSEA (https://doi.org/10.1073/pnas.0506580102, http://software.broa 138-dinstitute.org/gsea/index.jsp) to explore the enriched KEGG pathway between C1 (the sepsis subtype) and C2 (the control). The baseline gene set was 5, and the utmost gene set was 5,000 according to the expression profiling and phenotypic group, with 1000 resamples, FDR < 0.1, and adjusted *P* < 0.05 being significant statistically.

### Immunity cell populations and immunity checkpoint genes analysis

Based on our expression profiles, the CIBERSORT technique^25^ was adopted to examine the differential score of immunity cell populations between C1 and C2 using the R software package IOBR. The CIBERSORT includes 22 immune cell subtypes, and the linear regression approach was used to perform deconvolution analysis on the homo immunity cell subclass expression array. Subsequently, On the foundation of the expression profiling data, a differential analysis of immune checkpoint gene expression was accomplished on C1 and C2.

### Correlation analysis of hub-FRG and immunity cell populations and immunity checkpoint gene expression

Based on our expression patterns, Pearson's correlation coefficient analysis was conducted, respectively, on Hub-FRG and immune cells and Hub-FRG and immune checkpoint genes using our expression profiles and the Corrplot tool in R. The results are displayed as a heatmap.

### Development of an animal model of sepsis

To determine the differential expression of Hub-FRGs in different sepsis, we created respectively two kinds of septic animal models of G** + **infection and G- , Staphylococcus aureus strain (*S.aureus*, ATCC29213, USA) and Escherichia coli strain (*E.coli*, ATCC25922, USA). In brief, healthy male C57BL/6 mice (CharrlesRiver, 4 to 5 weeks of age, 14 ± 1 g) induced sepsis by tail vein injection of *S.aureus* 100 ul (1.25 cfu/ml) and *E.coli* 100ul (1.25 cfu/ml). Before this study, the room temperature was 20–23 degrees Celsius, the relative humidity ranged from 40 to 70%, there was a 12 h day/night cycle, and there was unrestricted access to drink and food. After 1 day of the model, mice were euthanized by utilizing chloral pentobarbital with i.p., and peripheral blood, heart, liver, lung, and kidney tissues were taken.

### Bacteria-burden experiment

The bacterial count of septic mice's hearts, lungs, liver, and kidneys—essential organs—was observed. Simply put, tissue samples were fragmented with a crusher (FastPep-24M5G), 500 ul of sterile PBS was given to the homogenate, and 100 ul of the homogenate was evenly placed in solid bacteria enhancement culture medium (Thermerfei Colombian subtype, stock number PB0123A, specification 10 × 90 mm), 37 °C and bacterial colony statistics were performed after 12 h of incubation.

### Biochemical detection

A small animal automated biochemical analyzer was used to perform peripheral blood biochemical detection of essential organ function (cardiac, hepatic, and renal) at 24 h following the sepsis model. The mouse jugular vein blood was obtained after an effective anesthesia (2% pentobarbitone, 45 mg/kg; peritoneal injection, pi), followed by centrifugation (1200 g, 4 °C, 10 min) to extract the light yellow serum, which was used for biochemical analysis by Small Animal Bioreagent chips LDH (#011,719, IDEXX), BUN (#012,133, IDEXX), CK (#010,838, IDEXX), and ALT (#011,052, IDEXX).

### Hematoxylin and eosin staining (HE)

After fixing mouse tissue samples in 4% paraformaldehyde solution for 24 h, they were dehydrated, transparent, wrapped in paraffin, cut into 4 μm sections, and dyed using hematoxylin–eosin (HE). A microscope was used to observe the heart, lung, liver, and kidney tissue lesions.

### ATP assay

A luciferase kit (ATP Assay Kit, Beyotime Biotech, China) was employed to test the ATP levels in the various organs of the sepsis model mice. In brief, the mouse heart, lung, liver, and kidney were lysed and centrifuged using lysis buffer (12,000 g, 4 °C, 5 min). Then, the upper liquid was instantly mixed with an equivalent volume of working solution before being placed in a standard opaque 96-well plate and detected by chemiluminescence in a photo recording plate reader (Thermo, Varioskan LUX, USA).

### Western blot analysis

The hearts, lungs, liver, and kidneys of sepsis model mice were lysed and centrifuged using lysis buffer (12,000 g, 4 °C, 10 min). A BCA protein detection kit (Beyotime Biotechnology Co., China) was employed to measure the concentration of supernatant protein. SDS-PAGE was used to separate the whole protein and afterwards transport it to a PVDF (polyvinylidene fluoride) film. After 1 h of sealing with 5% skimmed milk powder, apply the first antibody: against GPX4 (1:800, Cohesion, UK), 4 °C overnight. Then, at room temperature, incubate the membrane with the detection antibody for about 1 h. Finally, the protein bands were identified using a near-infrared, two-color laser imaging system (Odyssey CLX) and examined utilising a density measuring device (ImageJ 1.8.0).

### Immunofluorescence

In a 60 °C oven, paraffin sections were dewaxed, and the antigen was heat-extracted for 3 min. It was blocked with 10% donkey serum protein for 1 h at 37 °C before being treated with the primary antibody over night at 4 °C, anti-GPX4 (1:100, Cohesion, UK). Secondary antibodies with fluorescent colors were treated with sections at room temperature in the dark for 1 h. DAPI was then used to stain the nucleus (1:200, Bilyun, China) for 10 min. The findings were obtained using a CKX53 microscope (Olympus Optics Limited), and examined using a fluorescence-intensity measuring device (ImageJ 1.8.0).

### Ethics certification

This article's animal experiments were conducted to the Guide for the Care and Use of Laboratory Animals and were authorized by the Institutional Animal Care and Use Committee of the Army Medical University (No. TMMU2012009). All animal experiments were implemented in accordance with the recommendations outlined in the ARRIVE guidelines. All methods were performed in accordance with the relevant guidelines and regulations.

### Statistical analysis

The R package (v 4.0.2) was used for statistical analyses, as well as IBM SPSS 26.0 software (USA) and GraphPad Prism 8.0 (USA). The mean standard deviation (SD) was used to show all the data. The Student *t* test and Wilcoxon signed-rank test were applied to compare differences between the two groups. One-way ANOVA was used to evaluate several groups. Pearson's correlation coefficient was applied to examine the links between the sample groups. Furthermore, ROC technology was adopted to assess the diagnostic effectiveness of the core gene, which was represented by the Area Under Curve (AUC). The sensitivity and specificity of the gene were calculated. When Youden's index was adjusted to its maximum value, the optimum gene cut-off value was attained. *P < *0.05 represents statistical significance.

## Result

### Identification of DE-FRGs in sepsis

**Figure 2:**
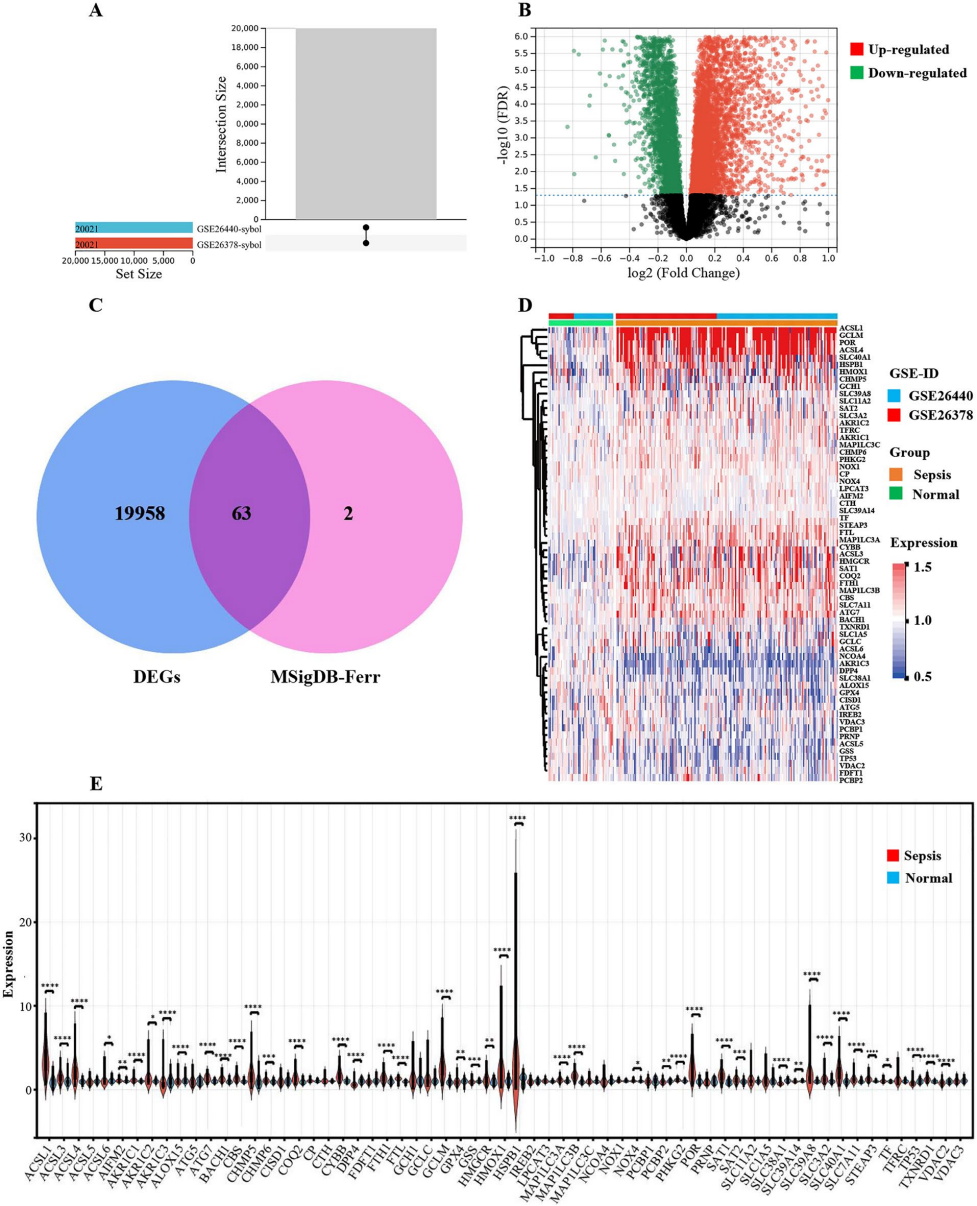
Identification of removing batches and DE-FRGs. (**A**) GSE26378 and GSE26440 were extracted from the batch and normalized, yielding a total of 20,021 genes. (**B**) Volcanic map visualization analysis revealed that 20,021 genes were differentially expressed, with 12,758 up-regulated genes and 7263 down-regulated genes. log2FC ≥ 1, adjusted *P* < 0.05. (**C**) A Venn diagram was used to identify 63 differently expressed FRGs as DE-FRGs. (**D**,** E**) The expression of 63 DE-FRGs was displayed using a heat map and a violin map.

After the debatch normalization treatment of GSE26378 and GSE26440 (Fig. 2A, Supplementary Figure 1), the differential expression analysis was performed, including 20,021 significantly differentially expressed genes, including 12,758 upregulated genes and 7,263 downregulated genes. log2FC ≥ 1, adjusted *P* < 0.05 (Fig. 2B). To explore differentially expressed FRG in sepsis, we extracted 65 FRG from MSigDB, a database based on human genes, and constructed them from location, function, metabolic pathways, and target binding. Following the intersection of DEGs and FRGs, 63 differentially expressed FRGs were identified as DE-FRGs (Fig. 2C). The expression of the 63 DE-FRGs was shown utilizing heatmap and violin plots (Fig. 2D,E).

### Functional enrichment analysis of the DE-FRGs

**Figure 3:**
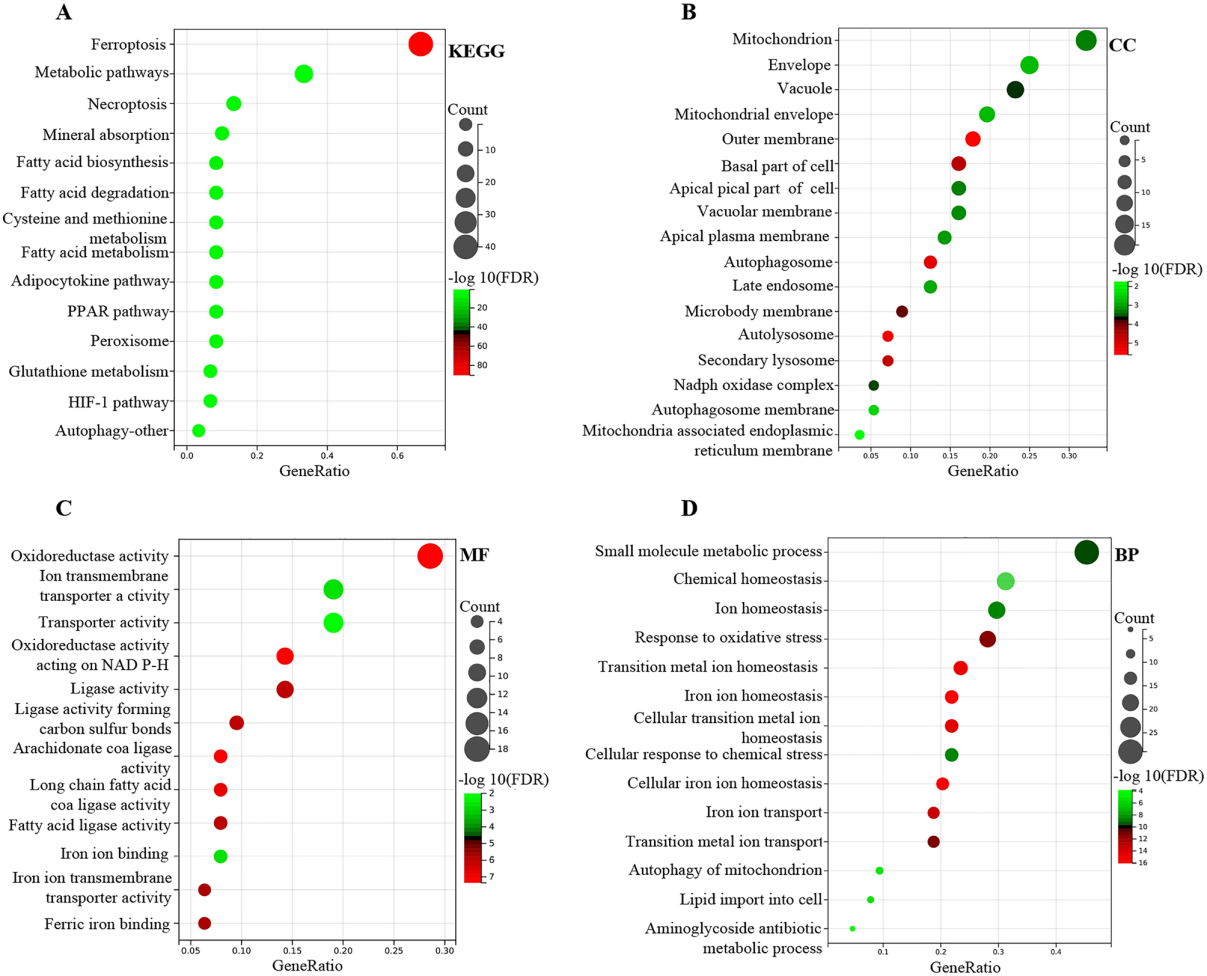
Bubble plot of GO and KEGG enrichment studies of DE-FRGs in sepsis specimens. The P value is shown by the progressively shifting hue, and the quantity of genes is denoted by the size of the black dots. (**A**) KEGG was mainly enriched in ferroptosis. (**B**) CC was mainly enriched in mitochondrion. (**C**) MF was mainly enriched in oxidoreductase activity. (**D**) BP was mainly enriched in small molecule metabolic processes.

Based on GO and KEGG, DE-FRGs' latent biological effects and pathways in both groups were investigated. According to the findings, KEGG was mainly enriched in ferroptosis, metabolic pathways, necroptosis, mineral absorption, and fatty acid production (Fig. 3A). CC (cell component) was significantly enriched in the mitochondrion, envelope, vacuole, mitochondrial envelope, and autophagosome (Fig. 3B). MF (molecular function) was highly enriched in oxygenate activity, ion transport transporter activity, transporter activity, iron ion binding, and iron ion transport transporter activity (Fig. 3C). BP (biological process) was highly enriched in small molecule metabolic processes, ion homeostasis, response to oxidative stress, iron ion homeostasis, and transition metal ion homeostasis (Fig. 3D). Obviously, these DE-FRGs were enriched in ferroptosis, mitochondria, autophagosomes, and oxidoreductase activity.

### PPI networks and core-FRG identification

**Figure 4:**
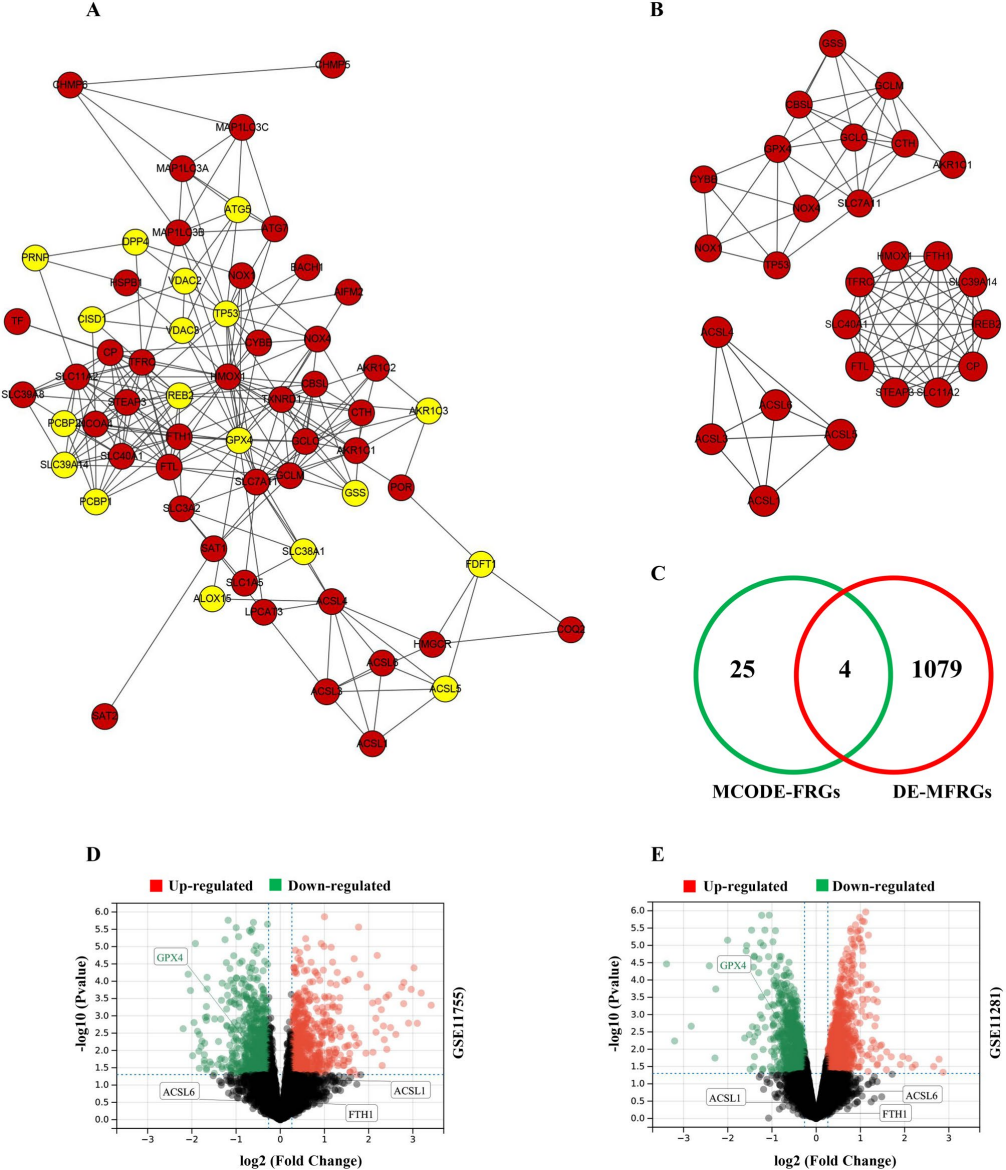
Construction of the PPI network and the MCODE module, and the acquisition and verification of Core-FRGs. (**A**) The DE-FRGs PPI network was generated using the Cytoscape tool and includes 61 nodes and 232 edges. (**B**) 3 MCODE-FRGs model were extracted. (**C**) MCODE-FRGs cross with DE-MFRGs to obtain 4 Core-FRGs (GPX4, ACSL1, ACSL6, FTH1). (**D**, **E**) Volcanic plots verified the expression of GPX4, ACSL1, ACSL6, and FTH1 in GSE11755 and GSE11281, respectively (adjusted *P* < 0.05, log2FC ≥ 1.2).

The PPI data of 63 DE-FRGs was taken from the String database, and the visual PPI network was built with the Cytoscape tool 3.9.1. There are 61 nodes and 232 edges in this network, and 45 genes were highly expressed, whereas 18 were underexpressed in 63 DE-FRGs (Fig. 4A). Three MCODE modules were obtained using the MCODE plug-in (Fig. 4B). In total, 29 MCODE genes were obtained, including STEAP3, FTL, SLC40A1, SLC39A14, HMOX1, IREB2, SLC11A2, FTH1, CP, TFRC, TP53, NOX4, CTH, GCLM, SLC7A11, GCLC, GSS, GPX4, NOX1, CBSL, CYBB, AKR1C1, ACSL3, ACSL4, ACSL1, ACSL5, and ACSL6.

Among the 1136 MFRGs, 1083 differentially expressed mitochondrial-related function genes (DE-MFRGs) were found, with 474 upregulated and 609 downregulated. Next, four differential genes were obtained after the DE-MFRGs were crossed with the MCODE-FRGs (FTH1, GPX4, ACSL1, and ACSL6) (Fig. 4C). We define it as the Core-FRG (Table 1).

**Table 1:** Functional annotation of 4 core-FRGs selected.

Gene symbol	Full name	Function
ACSL1	Acyl-CoA synthetase long Chain family member 1	Long-chain-fatty-acid-CoA ligase 1 is an enzyme that activates long-chain fatty acids to produce cellular lipids and degrades them through oxidation Palmitoleate, linoleate, and oleate are preferred; Acyl-CoA synthase clan (698 aa)
FTH1	Ferritin heavy chain 1	Heavy-chain ferritin stores iron in a soluble, non-toxic, easily available state. Iron homeostasis is critical. It possesses ferroxidase activity. After oxidation, iron is taken up in the ferrous state and deposited as ferric hydroxides. It also aids in the transport of iron to the cells. Regulates iron absorption in growing renal capsule ocytes (via resemblance); pertains to the ferritin family (183 aa)
GPX4	Glutathione peroxidase 4	Phospholipid hydrogen peroxide glutathione peroxidase; protects the cells from membrane lipid peroxidation and death of cells. Normal sperm growth and male fertility require it. It might have a significant role in shielding mammals against the toxicity of lipid hydrogen peroxide. It is necessary for the growth of the embryo. Avoid the effects of radiation and oxidation. It is required for photoreceptor development and survival. It aids in the first response of T cells to viral and parasite infections by preventing iron cell apoptosis (cell death induced by iron-dependent buildup of lipid reactive oxygen species) and promoting T cell growth; it is a member of the glutathione peroxidase family (197 aa)
ACSL6	Acyl-CoA synthetase long chain family member 6	Long-chain fatty acid coenzyme A ligase 6 is a coenzyme A ligase that activates the production of cellular lipids from long-chain fatty acids and their breakdown by β-oxidation. It is involved in brain fatty acid metabolism, and the acyl coenzyme A generated may be employed specifically for brain lipid synthesis. It is a member of the ATP-dependent, AMP-bound enzymatic family (722 aa)

The expression of Core-FRG was validated using two microarray datasets (GSE11755 and GSE11281). The volcanic plot displays that 2,116 DEGs were found in GSE 11,755 (up = 895; down = 1,217), and 2,096 DEGs were found in GSE 11,281 (up = 1,070; down = 1,026). log2FC ≥ 1.2, adjusted *P* < 0.05). Among the four Core-FRGs, only GPX4 was significantly and uniformly downregulated in different sepsis data sets (Fig. 4D,E), indicating that low levels of GPX4 expression can predict different sepsis types. Therefore, we define it as a hub-FRG and conduct subsequent verification.

### Diagnostic performance of GPX4 in the sepsis training set and verification set

**Figure 5:**
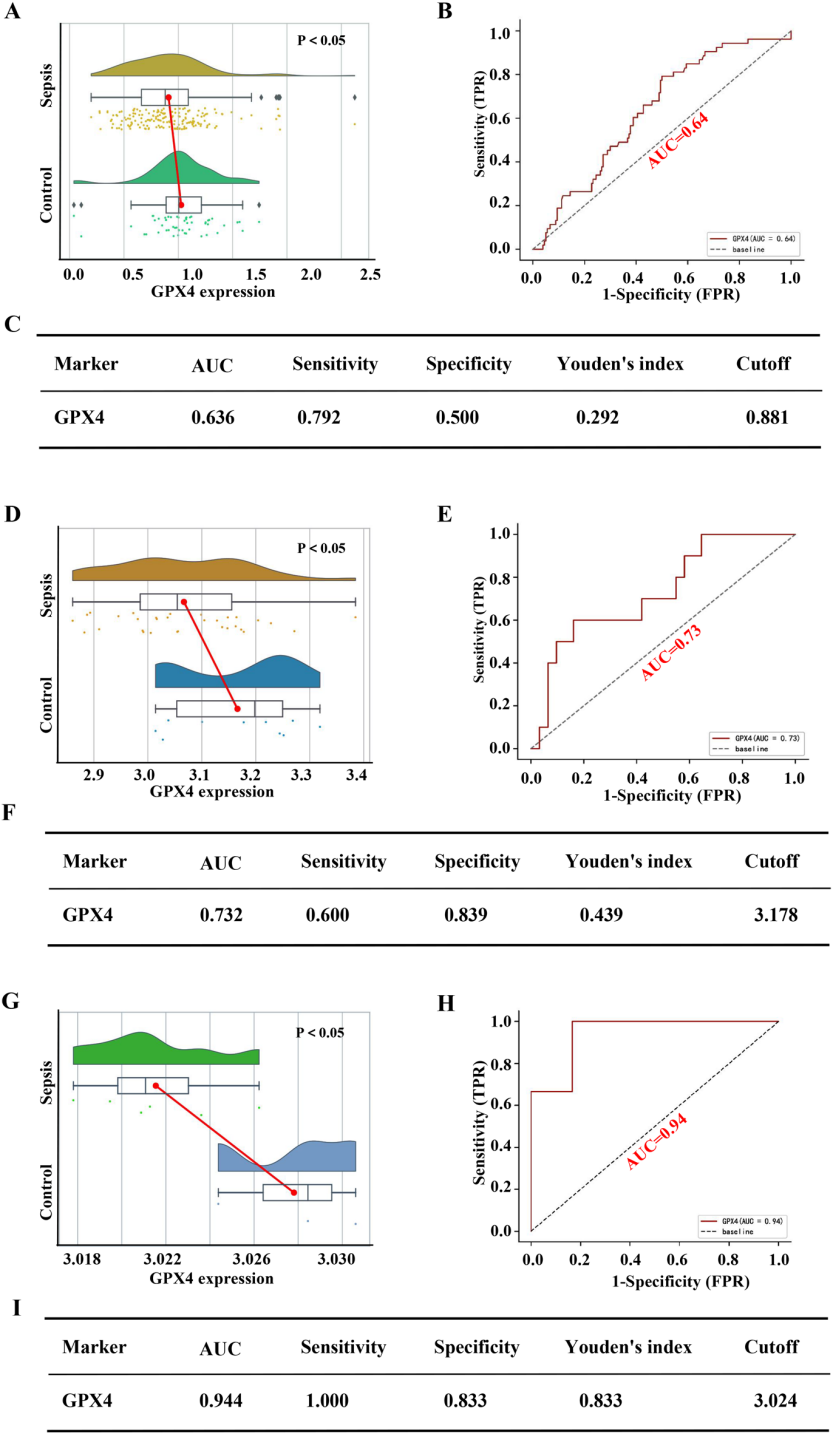
Performance of the hub-FRG diagnostic sepsis in the training and verification sets. Based on the training set expression profiles (GSE26378 and GSE26440): (**A**) The difference in GPX4 expression between the sepsis and control groups. (**B**) The ROC curve of patients with sepsis based on the GPX4 gene. (**C**) The diagnostic value of the hub-FRG in distinguishing the sepsis group from the control group. According to the validation set expression profile (GSE11755 and GSE11281): (**D**, **G**) The difference in GPX4 expression between the sepsis and control groups. (**E**, **H**) The ROC curve of people with sepsis based on the GPX4 gene. (**F**, **I**) The diagnostic value of the hub-FRG in distinguishing the sepsis group from the control group. AUC stands for Area Under the Curve; TPR stands for True Positive Rate; and FPR stands for False Positive Rate.

Figure 5 depicts the diagnostic value of GPX4 in sepsis. GPX4 was markedly downregulated in sepsis in the training set (GES 26,378 and GSE 26,440) relative to the control group (*P* < 0.05, Fig. 5A). The Area Under Curve (AUC) of the ROC of GPX4 in diagnosing sepsis was 0.64, with sensitivity and specificity of 0.79 and 0.5, respectively (Fig. 5B,C). Interestingly, the hub-FRG had better performance in the validation set (GSE11755 and GSE11281). GPX4 expression was significantly reduce in sepsis (*P* < 0.05, Fig. 5D,G). The AUC values were respectively 0.73 and 0.94 (Fig. 5E,H), its sensitivity value was respectively 0.6 and 1, and its specificity value was respectively 0.84 and 0.83. (Fig. 5 F,I). Evidently, these results suggest that GPX4 had a fine value for sepsis diagnosis.

### Identification of sepsis subtypes and enrichment analysis of GSEA for C1 and C2

**Figure 6:**
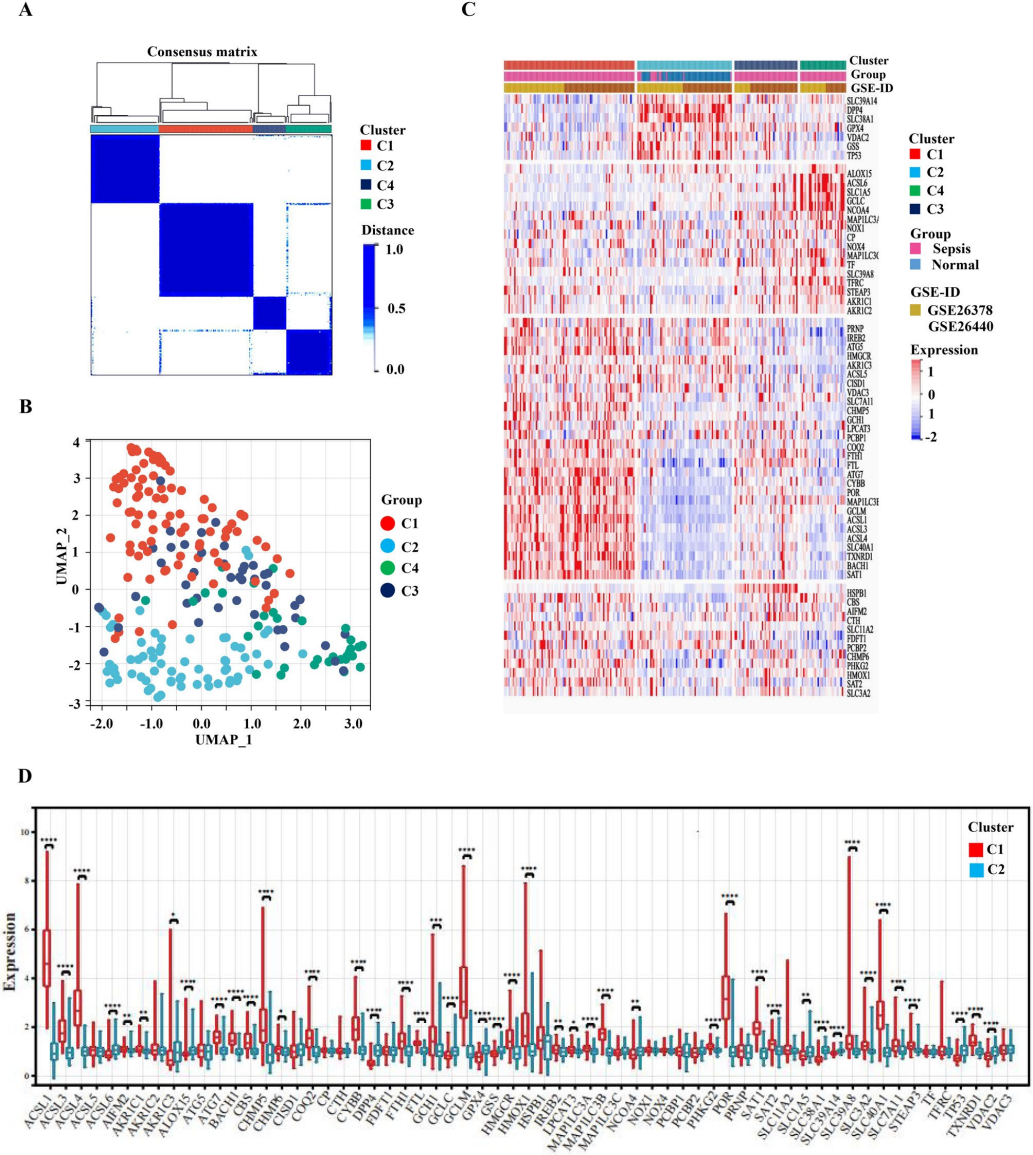
Cluster analysis and subtype identification based on DE-FRGs' expression profiles (**A**) The consensus matrix heatmap shows that DE FRGs can be grouped into four categories: C1 (N = 91), C2 (N = 66), C4 (N = 32), and C3 (N = 44). (**B**) The UMAP visualization confirmed clustering among different subtypes. (**C**) The heatmap depicts the various expression patterns of FRGs in four subtypes. (**D**) The box diagram shows that there are 43 FRGs with significant differences between C1 and C2, *P < 0.05, **P < 0.01, ****P < 0.0001.

According to the expression levels of DE-FRGs in the expression profile, the 233 samples were classified into four subgroups: C1 (N = 91 sepsis patients), C2 (N = 66, 53 control and 13 sepsis patients), C4 (N = 32 sepsis patients), and C3 (N = 44 sepsis patients) (Fig. 6A). From k = 2 to k = 10, the k = 2 cluster is the most constant (Supplementary Figure 2). The UMAP and heatmap studies indicated significant differences between C1 and the other subtypes, particularly C2 (Fig. 6B,C). Expression patterns of DE-FRGs change greatly between C1 and C2 (Fig. 6C,D). Compared with C2, 31 genes in C1 were significantly up-regulated (ACSL1, ACSL3, ACSL4, AKR1C1, ATG7, BACH1, CBS, CHMP5, CHMP6, COQ2, CYBB, FTH1, FTL, GCH1, GCLM, HMGCR, HMOX1, IREB2, LPCAT3, MAP1LC3A, MAP1LC3B, PHKG2, POR, SAT1, SAT2, SLC39A8, SLC3A2, SLC40A1, SLC7A11, STEAP3 and TXNRD1), 12 genes were significantly down-regulated (ACSL6, ALOX15, DPP4, GCLC, GPX4, GSS, NCOA4, SLC1A5, SLC38A1, SLC39A14, TP53 and VDAC2) (Fig. 6D).

Different pathways between C1 and C2 were compared by GSEA. The findings suggested that C1 was highly enriched in apoptosis, adipocytokine signaling path, lysosome, regulation of autophagy, Jak-stat signaling path, NOD-like receiver signaling path, RIG-I-like receiver signaling path, regulation of actin cytoskeleton, Toll-like receiver signaling path, PPAR signaling path, Fc epsilon RI signaling path, Fc gamma receiver mediated phagocytosis, and MAPK signaling pathway (Supplementary Figure 3).

### Analysis of immunity cell populations and immunity checkpoint for C1 and C2

**Figure 7:**
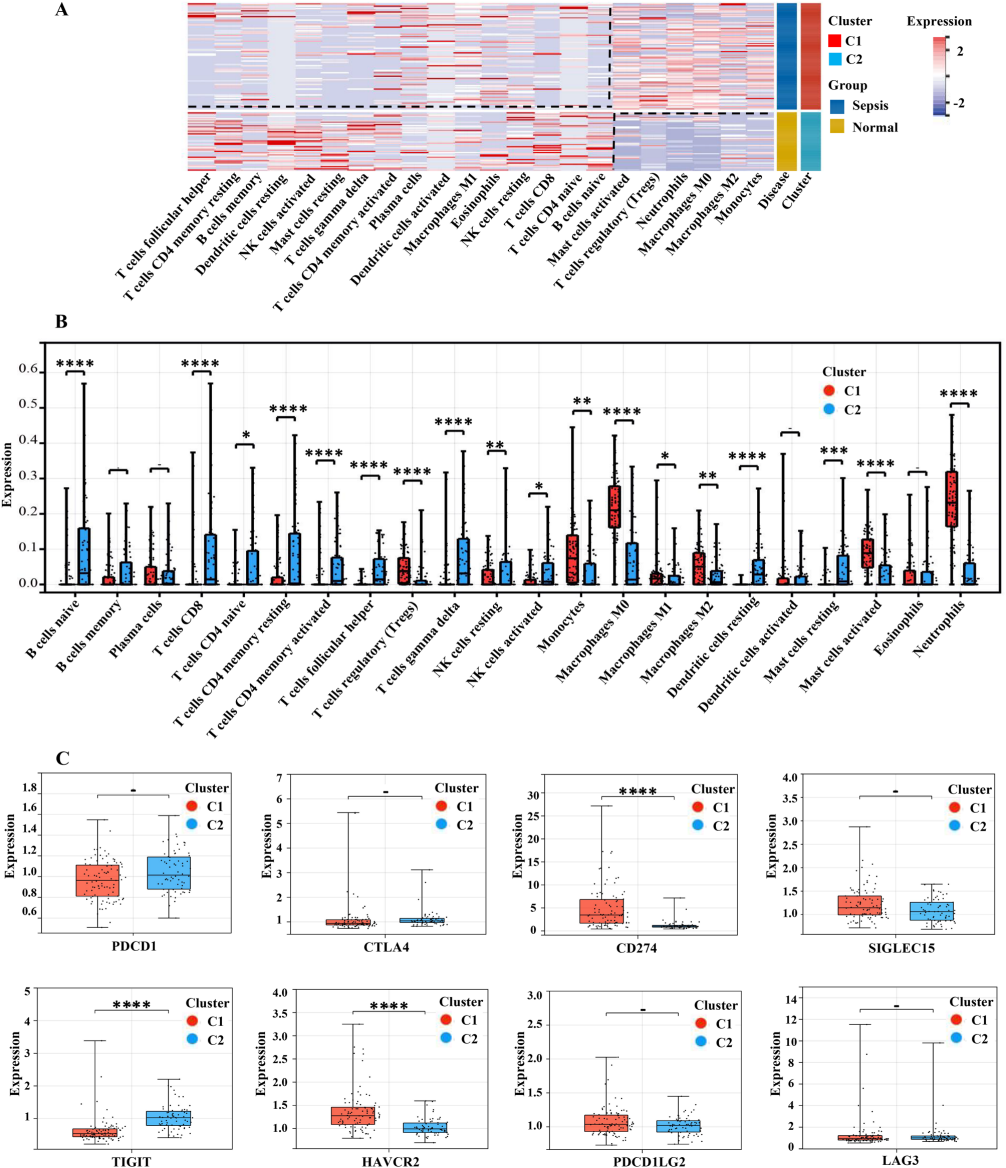
Characteristics of Sepsis subtype immuno cell populations and immunological checkpoint gene expression in peripheral blood. (**A**) The heat map displays the differences in immune cell expression patterns between Clusters 1 and 2. Blue represents low expression, whereas red represents strong expression. (**B**, **C**) The box graphs show the difference in immunity cell populations and immuno checkpoint expression between cluster 1 and cluster 2. *P < 0.05, **P < 0.01, ***P < 0.001, ****P < 0.0001.

The CIBERSORT tool was used to compute and assess diversities in the expression levels of the 22 immune cells in peripheral blood between C1 and C2 (Fig. 7A). There were significantly different patterns of expression in 17 immune cells (Fig. 7B). Compared to C2, the expression of seven types of immune cells was substantially higher in C1: mononuclear cells, macrophages-M0, macrophages-M1, macrophages-M2, activated mast cells, neutrophils, and Tregs. Besides, the expression levels of 10 kinds of immune cells, containing B cells, NK cells activated, T cells D4 naive, T cells CD8, T cells CD4 memory resetting, T cells CD4 memory activated, T cells gamma delta, Dendritic cells resetting, Mast cells resetting, and T cells follicular helper, decreased significantly.

Subsequently, we investigated the diversities in the expression amount of the eight immunity checkpoint genes between C1 and C2 (Fig. 7C). In contrast to the C2, a total of two immune checkpoint gene expression levels were significantly upregulated in the C1: CD274 (Programmed Cell Death 1 Ligand 1, PDL1) and HAVCR2 (hepatitis A virus cellular receptor 2; TIM3), and one expression level was significantly downregulated: TIGIT (T-cell immunoreceptor with Ig and ITIM domains, WUCAM, Vstm3, VSIG9).

### Hub-FRG (GPX4) correlation study with immunological cell populations and immunity checkpoints

**Figure 8:**
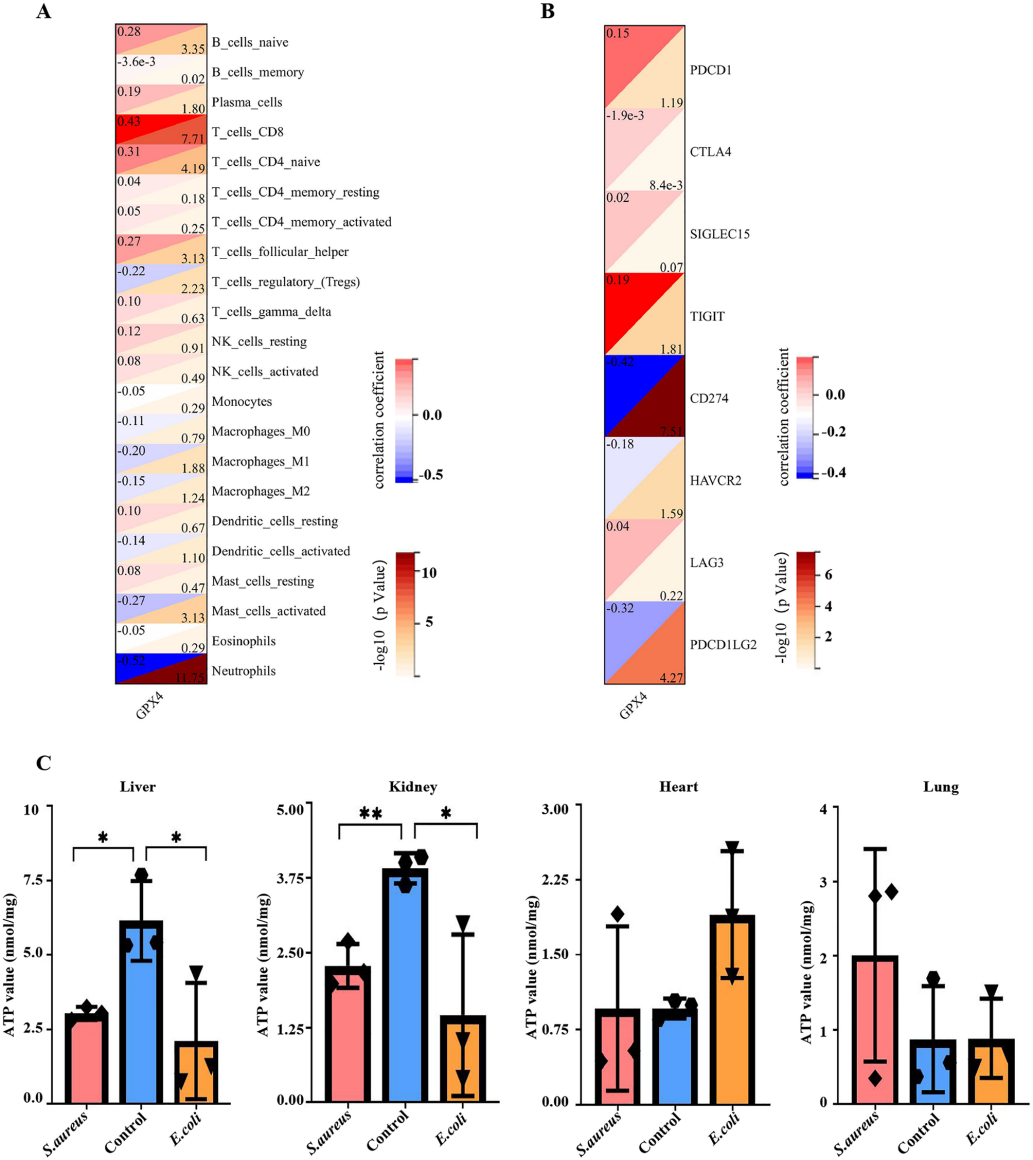
Correlation analysis between Hub FRG (GPX4) and immunity cells and immunity checkpoint genes; Detection of mitochondrial function in septic mice. (**A**) The heatmap displays the correlation between GPX4 and immunological cells, with neutrophils having the strongest correlation at r =  − 0.52 and − log10 *P* = 11.75, indicating a negative connection. T cells CD8 are followed by r = 0.43, − log10 *P* = 7.71, indicating a positive connection. (**B**) The correlation between GPX4 and the immunological checkpoint gene CD274 has the strongest correlation, r =  − 0.42, − log10 *P* = 7.51, indicating a negative link. PDCD1LG2 is ranked second with r =  − 0.32, − log10 *P* = 4.27, indicating a negative association. (**C**) ATP level measurement in tissues and organs showed that the ATP amount in the liver and kidney of the model group was significantly lower than the control, *P < 0.05. It indicated that mitochondria in liver and kidney tissues were seriously damaged.

The Pearson's correlation coefficient analysis of GPX4 and immune cell populations revealed that GPX4 was significantly associated with nine immune cells, with the highest negative Neutrophils correlation, r =  − 0.52, *P* < 0.0001, followed by T cells CD8, with a positive correlation, r = 0.43, *P* < 0.0001. Other intrinsic immune cells were shown to be negatively correlated (Macrophages M1, r =  − 0.20, *P* < 0.05; Mast cells activated, r =  − 0.27, *P* < 0.001), And other adaptive immune cells were positively correlated (T cells D4 naive, r = 0.31, *P* < 0.0001; B cells naive, r = 0.28, *P* < 0.001; T cells follicular helper, r = 0.27, *P* < 0.001; Plasma cells, r = 0.19, *P* < 0.05), except Tregs (r =  − 0.22, *P* < 0.01) (Fig. 8A).

Following that, a correlation analysis of GPX4 and immunological checkpoints revealed that GPX4 was markedly negatively correlated with CD274 (r =  − 0.42, *P* < 0.0001), PDCD1LG2 (r =  − 0.32, *P* < 0.0001), and HAVCR2 (r =  − 0.18, *P* < 0.05). There was a positive correlation with TIGI (r = 0.19, *P* < 0.05) (Fig. 8B).

### Sepsis induces multiple organs injury of mice

#### Organs invasion landscape of different strains in septic mice

In different sepsis models, heart, lung, liver, and kidney bacteria load experiments revealed that liver and kidney colonies were strongly colonized, and *S. aureus* was significantly higher than *E. coli* (*P* < 0.0001). In lung and heart colonies, there was less invasion (Supplementary Figure 4A,B).

#### Multiple-organ functional impairment in septic mice

To understand the impairment of essential organ function following sepsis, biochemical detection of heart, liver, and renal function in septic mice was accomplished. The results revealed that, compared to the control group, the model group's enzyme release from the liver (blood ALT) and kidney (blood BUN) was considerably higher (*P* < 0.05), with a statistical difference (Supplementary Figure 4C). CK and LDH, on the other hand, did not rise substantially (*P* < 0.05).

#### Sepsis causes pathological changes in multiple organs of mice

Histopathological changes in essential organs were observed under the microscope. HE staining was used to assess the damage to the heart, lungs, liver, and kidney. Compared to the control, the sepsis group's liver and kidney tissues were significantly injured, with edematous and necrotic liver cells, diminished liver plates, and a large number of inflammatory cells infiltrating locally. Renal tubule expansion, epithelial vacuolar degeneration, brush border endothelial cell separation, necrosis, and local inflammatory cell infiltration are all symptoms of renal tubule expansion. The group with *S.aureus* sepsis was even worse (Supplementary Figure 4D). The above indicates that the sepsis model was successfully constructed.

### Sepsis causes damage to mitochondrial function in multiple organs in mice

ATP levels in the hearts, lungs, liver, and kidneys of model mice were measured. The findings indicate that, when compared with the control, the amount of ATP detected in the liver and kidneys of the model group was considerably lower (*P* < 0.05) (Fig. 8C). These demonstrated that sepsis resulted in significant mitochondrial dysfunction in mouse liver and kidney cells.

### Changes in GPX4 expression levels in septic mouse multiple-organ tissues

**Figure 9:**
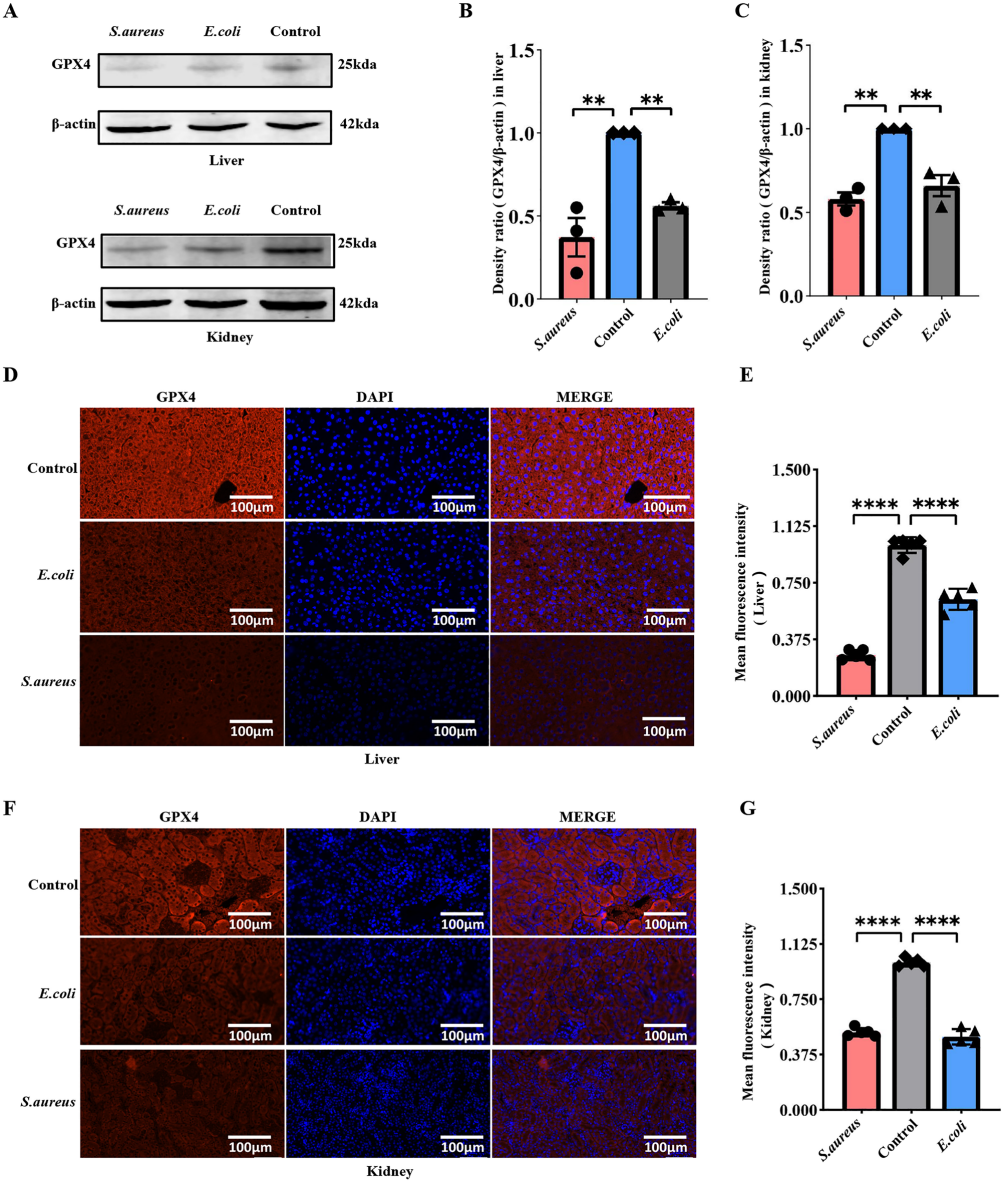
Expression level of GPX4 in the liver and kidney of sepsis mice (**A**) After sepsis mice were induced by *S. aureus* and *E. coli* for 24 h, the protein level of GPX4 in liver and kidney homogenates was detected by Western blot. (**B**, **C**) Densitometric analysis of correlation bands The expression of GPX4 protein in the liver and kidney of sepsis mice was remarkably lower than the control, **P < 0.01, respectively. (**D**, **F**) Immunofluorescence displayed that GPX4 was broadly expressed in normal liver and kidney tissues of mice, but significantly reduced in sepsis mice (DAPI: blue; GPX4: red; scale bar = 100 μm). (**E**, **G**) fluorescence statistical results (mean ± SD, n = 5), ****P < 0.0001 versus the control.

GPX4 expression was detected in major organs and tissues of various sepsis mice using Western blot (Wb) and immunofluorescence, respectively. The expression level of GPX4 in the liver and renal tissues of *S.aureus* sepsis and *E.coli* sepsis groups decreased considerably relative to the control in Wb (*P* < 0.01) (Fig. 9A,B,C, West blot results were from different parts of the same gel). Furthermore, consistent with Western blot, immunofluorescence revealed that GPX4 expression in the liver and kidney of septic mice was considerably lower than the control (*P* < 0.0001) (Fig. 9D,E,F,G). The findings indicate that sepsis-induced multiple organ function injuries are correlated with ferroptosis because GPX4 is frequently regarded as a star marker for ferroptosis.

## Discussion

Sepsis, induced by a severe infection, causes various organ malfunctions and threatens one's life^1,26^. Both children and adults can suffer from sepsis^27,28^, and the incidence and fatality rate of sepsis in children is higher, particularly in undeveloped regions^4^. Recently, a large volume of research has repeatedly underlined the critical significance of ferroptosis in sepsis^29^. In this study, four children's sepsis data sets, including two training data sets (GSE26378 and GSE26440) and two validation data sets (GSE11755 and GSE11281), were selected from the GEO database to execute bio-informatics analysis. The results discovered that one hub-FRG (GPX4) was a valuable biomarker of diagnosis (Fig. 5) and played a critical role in the onset and progression of sepsis.

GPX4 (Glutathione Peroxidase 4) is a protein-coding gene. It is a member of the selenoprotein family, plays a crucial role in ferroptosis, and can be utilized as an indication of cell ferroptosis^30^. Its primary effect is to convert peroxides (such as R-OOH) to equivalent alcohol (R-OH)^10^. The Xc system transports cystine to synthesize glutathione (GSH) to maintain redox balance and suppress iron poisoning, while GSH can also cooperate with GPX4 to enhance peroxide reduction^31^. As a result, GPX4 makes an important contribution to the removal of lipid peroxides. Inactivation of GPX4 can disrupt the oxidative balance, limit its function in eliminating lipid peroxides, injure mitochondria, and induce ferroptosis^30^. Interestingly, Zhang J et al. found irisin can reduce AKI caused by acute ischemia renal damage by up-regulating GPX4^32^. Wei S et al. reported that exogenous irisin could promote the expression of GPX4 and suppress hepatic ferroptosis in septic mice^33^. Extracellular CIRP drives GPX4-induced ferroptosis, according to Shimizu J et al.^34^. In this paper, in order to verify the reliability of hub-FRG prediction in the multiorgan function impairment caused by sepsis, we successfully constructed two different sepsis models (Supplementary Figure 4) to analyze further and validate the changes in GPX4 expression level in different organs and tissues of sepsis mice. The results of the Western blot and tissue immunofluorescence revealed that the model group's GPX4 expression in the liver and kidney was significantly lower than that of the control group (Fig. 9). These above results demonstrate that the ferroptosis-related gene GPX4 is tightly correlated with multiple organ function damage induced by sepsis, which is in accordance with our prediction.

Furthermore, mitochondrial damage is a change in ferroptosis characteristics^35^ and is one of the primary causes of sepsis' poor prognosis^36^. Because mitochondria are the central source of ATP production, analyzing ATP levels can directly represent cell mitochondrial function. In this study, the activity of mitochondria in the heart, liver, kidney, and lung was measured using an ATP test. The results revealed that the ATP levels in the model group were lower than in the control group to varied degrees. However, it was most noticeable in the liver and kidney, with *P* < 0.05 (Fig. 8C). These findings supported the theory that ferroptosis is accompanied by mitochondrial functional damage.

Acknowledgedly, sepsis is closely related to immune regulation disorder^26^. Immune dysfunction may be one of the major factors that cause organ dysfunction or failure, or even death, in sepsis patients^37^. This study demonstrated that in children with sepsis, expression of the innate immune cells monocytes, macrophages M0, macrophages M1, macrophages M2, mast cells activated, and neutrophils were obviously up-regulated, whereas dendritic cells resting were significantly down-regulated (Fig. 7A,B). Interestingly, many investigations have found that innate immune cells express abnormally in sepsis. Asmaa et al. reported that (neonatal sepsis, n = 30, and control, n = 30), CD86+ monocytes in the sepsis (78.4%) were evidently higher than the control (24%) (*P* < 0.001)^38^. Eash KJ et al. found that CXCL1 was up-regulated in mouse sepsis, which could drive neutrophils to release into the blood^39^. Bouras M et al. discovered that the number of interstitial DCs in sepsis patients' peripheral organs was much lower than in non-sepsis patients^40^.

Moreover, this research found that except for up-regulated T cell regulation (Tregs), all others in acquired immunity were downregulated (B cells, CD8 T cells, D4 live T cells, CD4 memory resting T cells, CD4 memory activated T cells, follicular helper T cells, gamma delta T cells , activated NK cells ) in septic patients (Fig. 7B). Venet F et al. showed that following sepsis, the number of B cells, T cells, NK cells, and other lymphocytes reduced dramatically^41^, an essential feature of the adaptive immune response^41,42^. In patients with sepsis, reducing the persisting lymphoid layer increases the risk of mortality and hospital infection^43,44^. In addition, Jensen et al. have shown that sepsis may induce and alter the phenotypic effect of CD8 T cells, which may weaken the control of Listeria monocytogenes infection and increase the chance of reinfection^45^. Li Z et al. observed that B cells, CD4 + T cells, T cells follicular helper, CD8 + T cells, and NK cells were considerably decreased in childhood sepsis^46^, which was consistent with the findings of our investigation.

Immune checkpoint analysis revealed that the expression of CD274 (PD-L1) and HAVCR2 (TIM) was increased evidently in sepsis relative to the control (Fig. 7C). PD-L1 is the focus of current immune checkpoint research. The abnormal increase of PD-L1 expression in sepsis patients may be the leading cause of immunosuppression^47^, which is now considered one of the leading causes of sepsis death^48^. PD-L1 expression rises on a range of cells during sepsis, such as neutrophils^49^, stromal cells, dendritic cells, macrophages, capillary endothelial cells^50^, and monocytes^51^. Furthermore, the level of PD-L1 expression in monocytes and neutrophils is associated with the prognosis and survival of sepsis patients^49,52^. Chang KC^53^ and Zhang Y^54^ discovered that using PD-1 or PD-L1 antibodies to treat animal models of bacterial and fungal-induced sepsis can improve overall survival. These offer a theoretical foundation for future clinical trials.

Notably, immune cells (B cells, T cells, Macrophages, and so on) themselves may also suffer ferroptosis, which is then recognized by immunity cells, resulting in a cascade of immunological reactions^55^. This study demonstrates that GPX4 was significantly tied with the expression levels of different immunity cells (Neutrophils, CD8T cells, CD4 nave T cells , Tregs, Macrophages M1, and Master cells activated) and immune checkpoint genes (CD274, TIGIT, HAVCR2, and PDCD1LG2) (Fig. 8A,B). These data implied that GPX4 was connected with immune modulation and can be a new latent immunotherapeutic target of pediatric sepsis. To summarize, GPX4 was a valuable biomarker for diagnosing sepsis and had a non-negligible role in the multi-organ functional impairment caused by sepsis-induced ferroptosis in kids.

Nevertheless, the research also contains several restrictions. First, consider the patient sample size constraint. Consequently, GPX4 research should be included in the broader sepsis queue. Second, this study only focused on the expression level of GPX4 in sepsis rather than its function in vivo or in vitro. As a result, further exploration into ferroptosis in children with sepsis should be required in the future.

## Conclusion

In summary, this paper demonstrated that the ferroptosis-related gene GPX4 played a crucial role in the progression of multiple organ damage in childhood sepsis, was closely related to immunity cell populations and immunity checkpoint regulation, and was a biomarker of crucial diagnostic value and a latent immunotherapy target for this disease.

## Supplementary Information

Supplementary Information 1.

Supplementary Information 2.

## Data Availability

Datasets analysed in this study are available from GEO database(http://www.ncbi.nlm.nih. gov/geo/). Open-tier datasets not requiring access approval are available for download via GEO (accession number GSE26378: https://www.ncbi.nlm.nih.gov/geo/query/acc.cgi?acc=GSE26378; accession number GSE26440: https://www.ncbi.nlm.nih.gov/geo/query/acc.cgi?acc=GSE26440; accession number GSE11755: https://www.ncbi.nlm.nih.gov/geo/query/acc.cgi?acc=GSE11755; accession number GSE11281: https://www.ncbi.nlm.nih.gov/geo/query/acc.cgi?acc=GSE11281). The FRGs dataset is available for browsing via MSigDb databank (https://www.gsea-msigdb.org/gsea/msigdb/), and the MFRGs dataset is available from Mitocarta 3.0 online data base (https://www.broadinstitute.org/mitocarta).
