# Supplementary material for: GPX4 is a key ferroptosis biomarker and correlated with immune cell populations and immune checkpoints in childhood sepsis
Source: Sci Rep. 2023 Jul 13;13:11358. doi: 10.1038/s41598-023-32992-9 (PMC10345139; doi:10.1038/s41598-023-32992-9)
Supplement: Supplementary file 1 — Supplementary Information 1. [file 41598_2023_32992_MOESM1_ESM.pdf]

Supplementary Material

Supplementary Figures

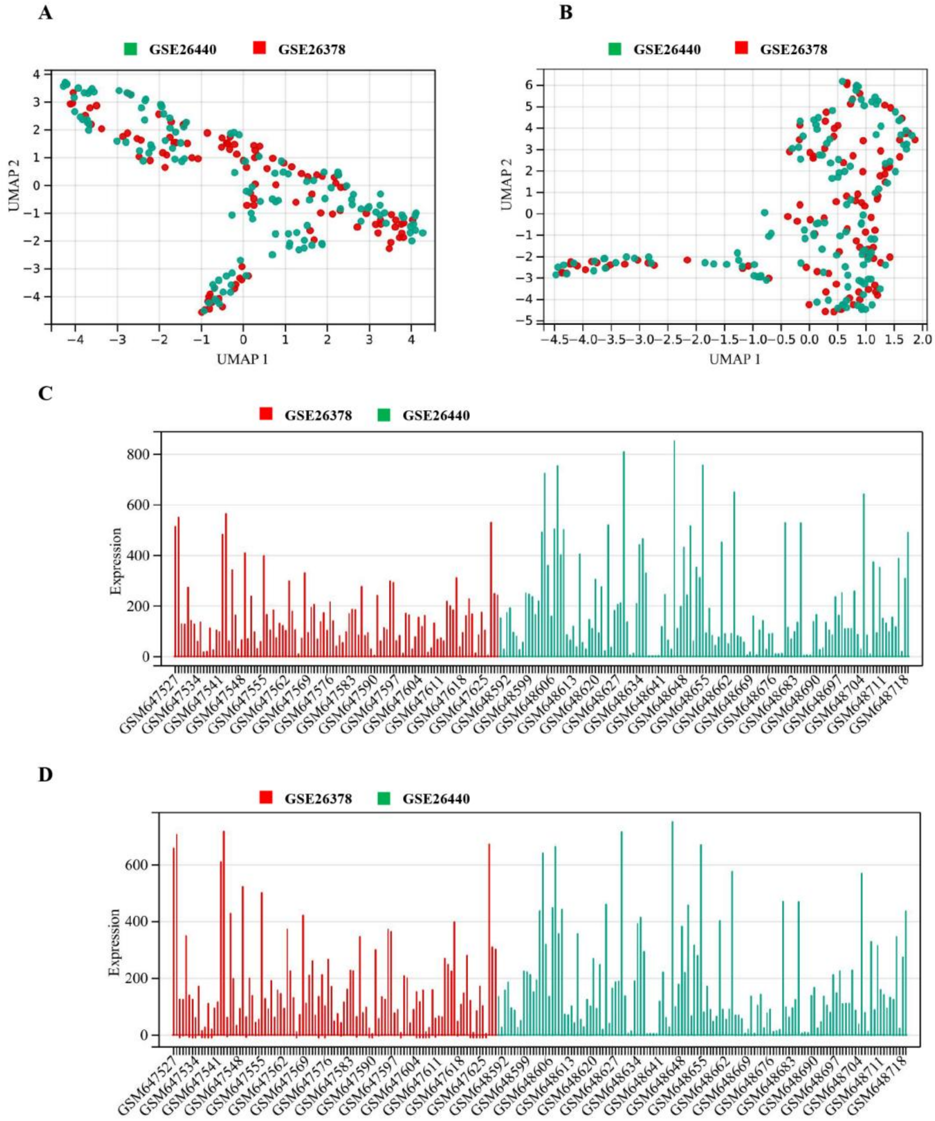

Supplementary Figure 1 The UMAP diagram shows that **(A)** before removing the batch effect, the samples from each data set are clustered together, respectively, and **(B)** after removing the batch effect, the samples from each data set are intertwined with each other, indicating that the batch effect is removed better. The box plot shows that **(C)** the sample distribution of each data set is considerably different before removing the batch effect, demonstrating that there is a batch effect. **(D)** After the batch effect is removed, the data distribution within each data set tends to be consistent, with the median on one line.

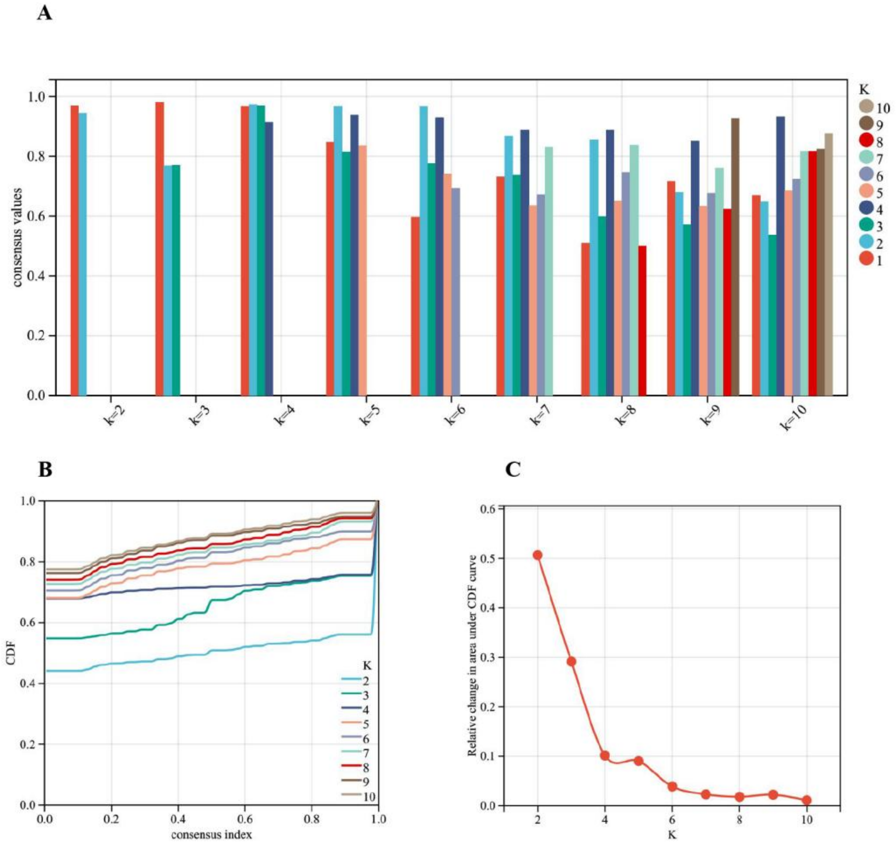

Supplementary Figure 2 Consensus clustering of FRGs. **(A)** displays the number of clusters inside the group with the highest average consistency as K=2, while the number of clusters with the second highest consistency is K=4. **(B-C)** show the consensus clustering cumulative distribution function of K=2 to K=10.

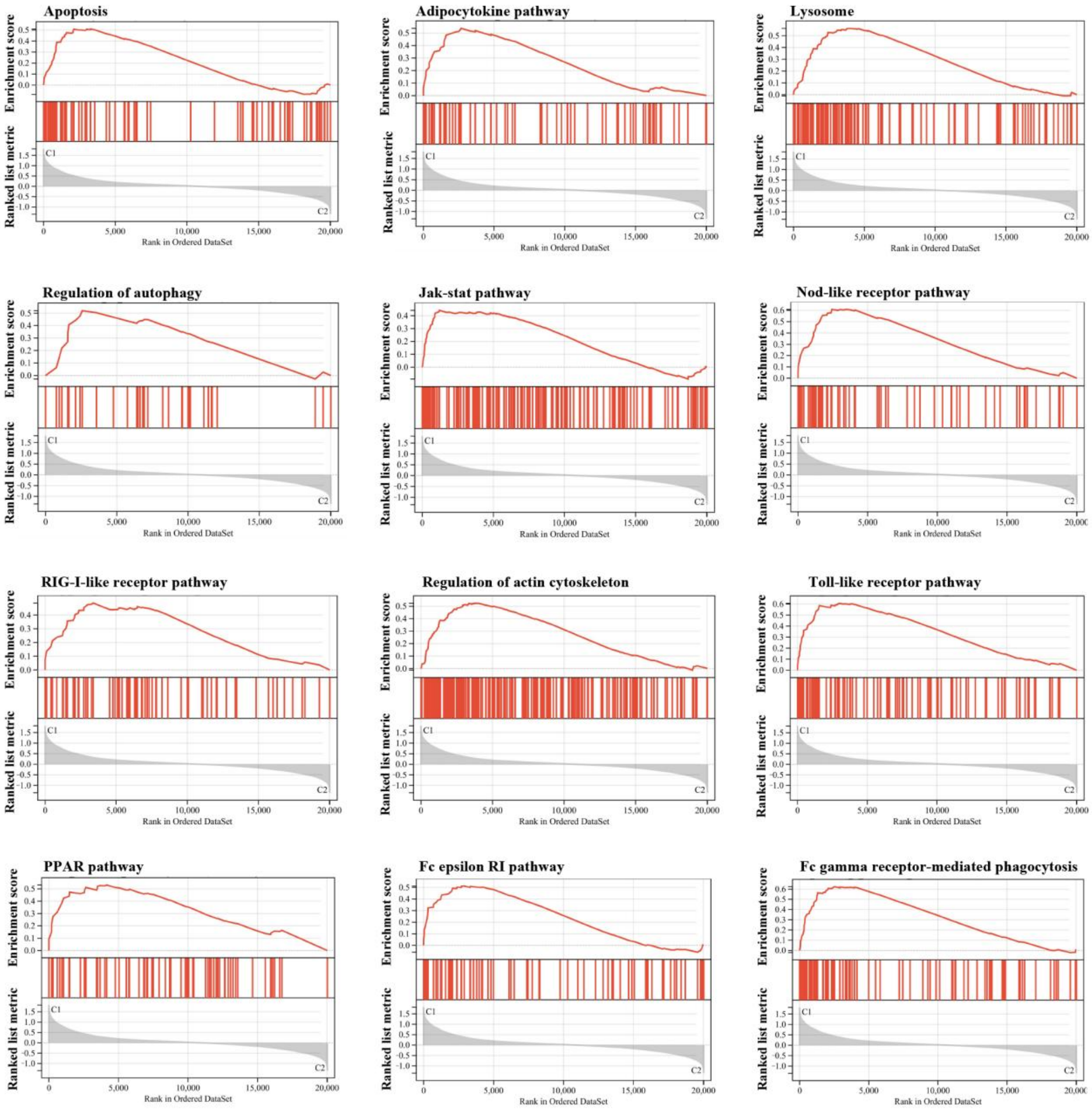

Supplementary Figure 3 Gene set enrichment analysis. GSEA in C1 and C2. (NOM) P＜0.05, FDR＜10%.

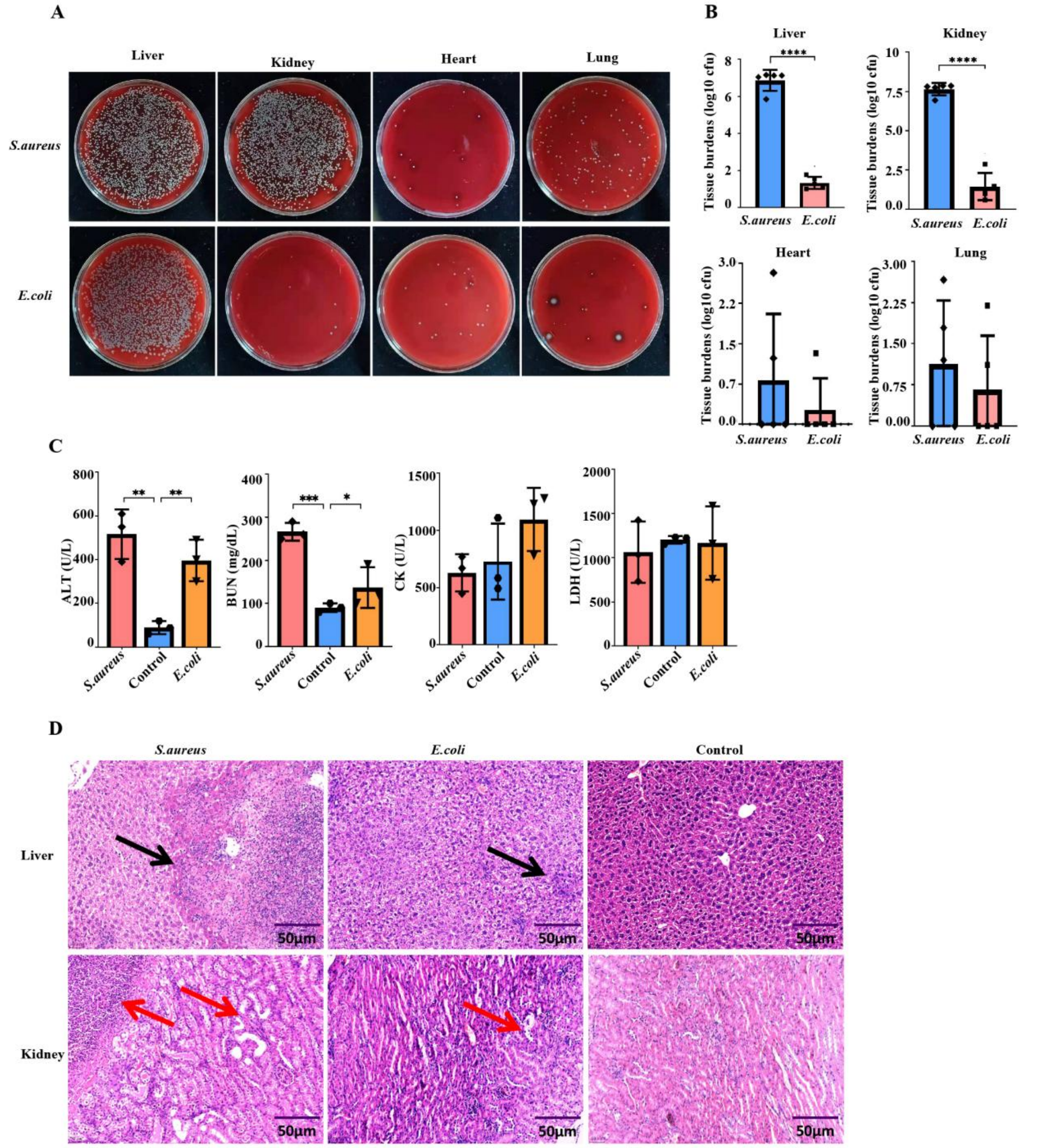

Supplementary Figure 4 The landscape of colonization and invasiveness of the *S. aureus* strain and the *E. coli* strain in major organs of septic mice, and the detection of the function and histopathology of important organs in septic mice. **(A)** The bacteria burden experiment showed that colony colonization and cloning occurred in the mice with *S. auerus* sepsis and in the liver, kidney, heart, and lung of the *E. coli* group. **(B)** According to the colony clone statistics in the liver, kidney, heart, and lung of model mice, the colony clone and invasiveness in the liver and kidney of *S. aureus* sepsis model mice were evidently higher than that of the *E. coli* group, ****P＜0.0001. **(C)** Biochemical test of small animals 24 hours after the model. Compared with the

control group, *S. aureus* and *E. coli* induced a significant increase in the release of liver ALT and kidney BUN in the peripheral blood of sepsis mice, indicating that liver and kidney functions were seriously injured, $^{*}P < 0.05$, $^{**}P < 0.01$, $^{***}P < 0.001$. **(D)** HE staining was used to detect histopathological changes. Representative liver and kidney tissue sections were stained with HE and magnified (Scale bar = 50 μm). The black arrow points to the site of liver injury; the red arrow points to the site of kidney injury.
