# Supplementary material for: GPX4 is a key ferroptosis biomarker and correlated with immune cell populations and immune checkpoints in childhood sepsis
Source: Sci Rep. 2023 Jul 13;13:11358. doi: 10.1038/s41598-023-32992-9 (PMC10345139; doi:10.1038/s41598-023-32992-9)
Supplement: Supplementary file 2 — Supplementary Information 2. [file 41598_2023_32992_MOESM2_ESM.pdf]

(1)West-blot original picture of GPX4 expression level in organs

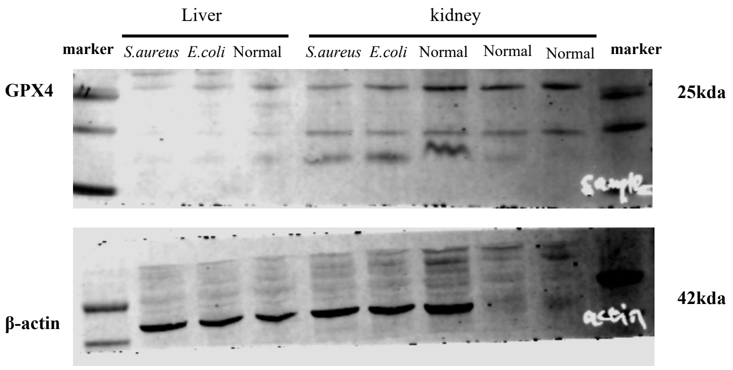

The above West blot results were from different parts of the same gel.

(2)West-blot original picture of GPX4 expression level in organs

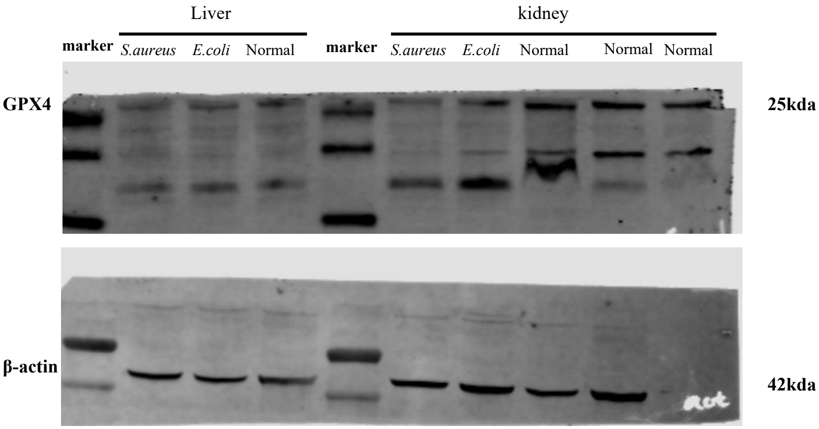

The above West blot results were from different parts of the same gel

(3)West-blot original picture of GPX4 expression level in organs

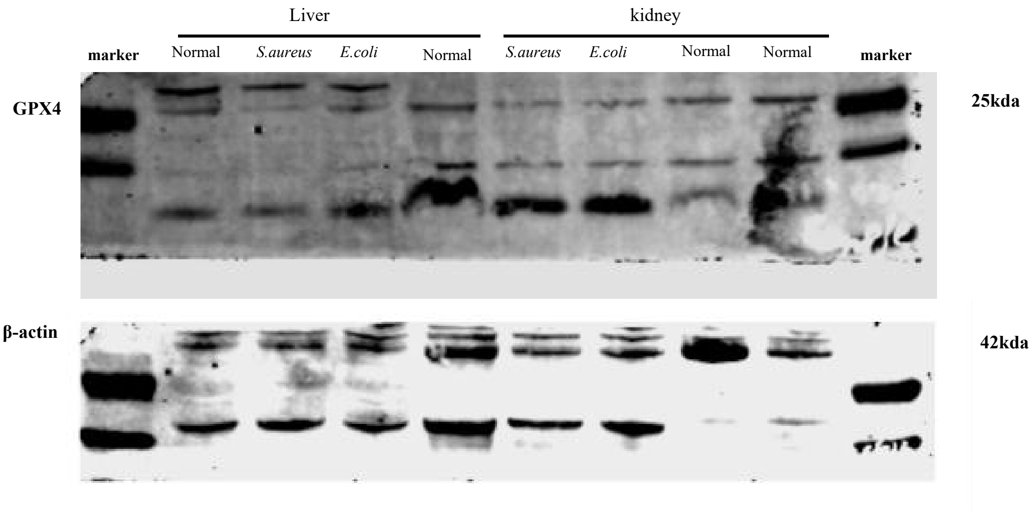

The above West blot results were from different parts of the same gel.
